# Analysis of the Olive Fruit Fly *Bactrocera oleae* Transcriptome and Phylogenetic Classification of the Major Detoxification Gene Families

**DOI:** 10.1371/journal.pone.0066533

**Published:** 2013-06-18

**Authors:** Nena Pavlidi, Wannes Dermauw, Stephane Rombauts, Antonis Chrisargiris, Thomas Van Leeuwen, John Vontas

**Affiliations:** 1 Department of Biology, University of Crete, Heraklion, Crete, Greece; 2 Department of Crop Protection, Ghent University, Ghent, Belgium; 3 Department of Plant Biotechnology and Bioinformatics, Ghent University, Ghent, Belgium; International Atomic Energy Agency, Austria

## Abstract

The olive fruit fly *Bactrocera oleae* has a unique ability to cope with olive flesh, and is the most destructive pest of olives worldwide. Its control has been largely based on the use of chemical insecticides, however, the selection of insecticide resistance against several insecticides has evolved. The study of detoxification mechanisms, which allow the olive fruit fly to defend against insecticides, and/or phytotoxins possibly present in the mesocarp, has been hampered by the lack of genomic information in this species. In the NCBI database less than 1,000 nucleotide sequences have been deposited, with less than 10 detoxification gene homologues in total. We used 454 pyrosequencing to produce, for the first time, a large transcriptome dataset for *B. oleae*. A total of 482,790 reads were assembled into 14,204 contigs. More than 60% of those contigs (8,630) were larger than 500 base pairs, and almost half of them matched with genes of the order of the Diptera. Analysis of the Gene Ontology (GO) distribution of unique contigs, suggests that, compared to other insects, the assembly is broadly representative for the *B. oleae* transcriptome. Furthermore, the transcriptome was found to contain 55 P450, 43 GST-, 15 CCE- and 18 ABC transporter-genes. Several of those detoxification genes, may putatively be involved in the ability of the olive fruit fly to deal with xenobiotics, such as plant phytotoxins and insecticides. In summary, our study has generated new data and genomic resources, which will substantially facilitate molecular studies in *B. oleae*, including elucidation of detoxification mechanisms of xenobiotic, as well as other important aspects of olive fruit fly biology.

## Introduction

The olive fruit fly, *Bactrocera oleae* (Diptera: Tephritidae) is the most important pest of olive orchards worldwide. The fly lays its eggs in the olive fruit and the developing larvae tunnels through the olive, feeding on the fleshy mesocarp, a plant tissue with high content of phenolic compounds and phytotoxins. The infestation severely affects the quality (up to 80%) and value of the olive oil and cause the rejection of entire harvests of table olives which become unsuitable for consumption [1]. In Greece, 30–35% economic losses due to *B. oleae* have been recorded, and the annual cost for its control exceeds 2 M euros.

The control of *B. oleae* has been based on use of traps, sterile insect techniques (SIT) and primarily insecticides. An improved genetic method, Release of Insects containing a Dominant Lethal (RIDL), was recently developed [2], however, regulation issues restrict its wide application at present. The intense use of insecticides, as baits or cover sprays, has resulted in the selection of resistance which negatively impacts on our ability to control *B. oleae* [3]. The elucidation of insecticide resistance mechanisms at the molecular level, in light of the development of tools for sustainable control, has been achieved in some cases in *B. oleae* and closely related Tephritidae species (reviewed in [3]). Modified acetylcholinesterase (MACE) resistance mechanism has been studied extensively in *B. oleae* [3], and specific mutations which reduce sensitivity to organophosphate insecticides have been identified and characterized [4], [5]. Target site resistance against spinosad was recently elucidated in the oriental fruit fly *Bactrocera dorsalis*, where truncated transcripts of nicotinic acetylcholine subunit gene Bdα6 were strongly implicated in the phenotype [6]. The study of metabolic resistance, *i.e.* mechanisms that increased rates of insecticide detoxification and thus compromises the effective dosage of the insecticide that reaches the target, has not kept similar pace. Detoxifying enzymes (such as cytochrome P450s (P450s), carboxyl/choline esterases (CCEs), and glutathione S-transferases (GSTs) have been associated with *B. oleae* insecticide resistance phenotypes [3]; however, the analysis of the mechanism at the molecular level has been hampered by the lack of genomic information and the complexity of detoxification gene families and pathways involved in resistance. These metabolic enzymes might be among the major weapons of *B. oleae* larvae to cope with phytotoxins and phenolic compounds present in olive flesh, in line with the adaptation mechanisms operating in other insect species [7], [8].

A study which investigated the interaction between *B. oleae* and olive was recently conducted from the plant perspective [9]. It revealed that the olive response to *B. oleae* larvae, its most damaging biotic stressor, resulted in the induction of genes implicated in the production of Reactive Oxygen Species (ROS), the activation of different stress response pathways and the production of compounds involved in direct defense against phytophagous larvae.

*B. oleae* is a strictly monophagous species and feeds, in contrast to the majority of fruit flies, on fresh instead of decaying substrates. The mechanism that *B. oleae* manages to overcome olive plant defense and utilize the fruit flesh has not been elucidated yet. It has been associated with symbiotic bacteria [10], which however only facilitate the development of the larvae in green olives, but not more mature ones.

To date (April 2013), only 802 *B. oleae* nucleotide and 876 protein sequences have been deposited in the NCBI database (http://www.ncbi.nlm.nih.gov), including less than 10 detoxification gene homologues in total. This data set is clearly not sufficient for the investigation of metabolism-based insecticide resistance mechanisms, or the study of molecular interactions between the olive and the fruit fly *B. oleae*. Here we report, for the first time, the use of 454-pyrosequencing technology to characterize the *B. oleae* (pooled stages) transcriptome and the identification and phylogenetic classification of a large number of genes potentially encoding detoxification enzymes.

## Results and Discussion

### 454 FLX Titanium Sequencing and Assembly

A library of pooled life stages of *B. oleae* were sequenced, using 454 pyrosequencing, in a single run on a picotiterplate (PTP). This resulted in 482,790 aligned reads with an average read length of 421 nucleotides and a total of 147,882,767 bases (Table 1). These reads were assembled into 14,204 contigs. More than 60% of the contigs (8,630 contigs) were larger than 500 base pairs (bp), with a total of 8,675,718 bases. The average contig size was 1,005 bp and largest contig size was 6,318 bp. The remaining contigs (5,574, 39.25%) ranged between 100–500 bp with a total of 10,240,327 bases. 126,383 reads could not be assembled and were classified as singletons while 363,905 and 11,980 reads were categorized as repeats and outliers, respectively. Compared to the previously reported *B. oleae* transcriptome dataset, consisting of 195 ESTs only and derived by single pass sequencing of a *B. oleae* adult cDNA library [11], our 454 pyrosequencing represents a substantial expansion to the genomic resources available for this species.

**Table 1:** Summary of run statistics and assembly.

Run statistics	
Total number of reads	1,012,155
Number of aligned reads	482,790
Average aligned read length	421 nucleotides
Total number of aligned bases	147,882,767
**454-Newbler Assembly**	
Number of full assembled reads	414,125
Number of partial assembled reads	68,529
Number of Singletons	126,383
Number of Repeats	363,905
Number of Outliers	11,980
Number of Too short reads	27,233
**Large contigs (>500** **bp)**	
Number of contigs	8,630
Number of bases	8,675,718
Average contig size	1,005
N50* Contig Size	1,087
Largest contig size	6,318
**All contigs (>100** **bp)**	
Number of contigs	14,204
Number of bases	10,240,327

* size above which 50% of the assembled sequences can be found.

### Homology Searches

**Figure 1:**
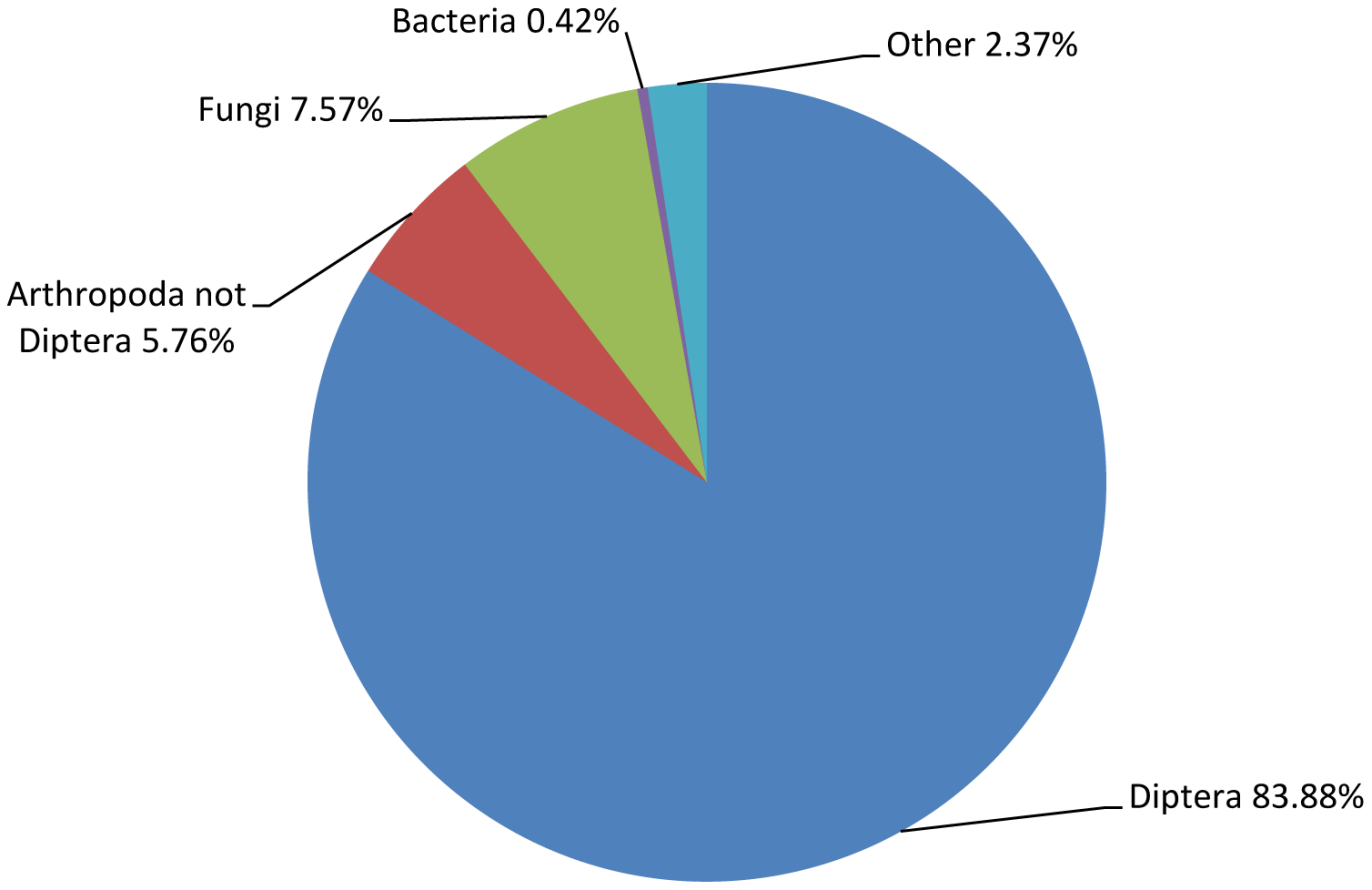
Analysis of the distribution of the 8,129 top blast hits obtained by blast against the nr database (NCBI). Percentage distribution within different taxonomic groups.

A total of 8,129 sequences of all contigs (65.36%) from the *B. oleae* transcriptome returned an above cut-off blast hit to the NCBI non-redundant protein database (Table S1). When an e-value cut-off of 1E^−3^ was used for blastx, 7,368 (59.24%) blast results were obtained, while when the e-value cut-off 1E^−10^ was used for blastn, 760 (6.12%) additional blast results were obtained. Blast statistics are presented in Figure S1. 83.88% (6,819 sequences) of the top blast hits correspond to Diptera, 5.75% (468 sequences) to other Arthropoda (except Diptera), 7.56% (615 sequences) to Fungi, 0.42% (34 sequences) to Bacteria and 2.37% (193 sequences) to other organisms (Figure 1). From the *B. oleae* contigs having their best blast hit with Fungi, 317 encoded for ribosomal RNA, 296 for hypothetical proteins, one for a transposase (contig07917) and one for a mitochondrial protein (contig03600). These 615 “fungi” contigs were considered as contamination and excluded from further analysis.

Analyzing the blast statistics more into detail revealed that the majority of *B. oleae* sequences returned its best blast hit for *Drosophila* species (5,299 sequences, 65.18%). Out of these species, *Drosophila virilis* returned the majority of blast hits (751 sequences, 9.23%) followed by *Drosophila willistoni* (696 sequences, 8.56%) and *Drosophila mojavensis* (672 sequences, 8.26%). Among other Diptera, 695 (8.54%) sequences had their best hit for *Glossina morsitans*, 94 (1.15%) for *Aedes aegypti* and 55 (0.67%) for *Culex quinquefasciatus*. The distribution is in accordance with that obtained by transcriptome analysis of the closely related Tephritidae species, *B. dorsalis* [12], where approximately 80% of the genes were most closely related to *Drosophila* homologues. Notably, only 65 (0.79%) sequences had their best match with *B. oleae* sequences subjected to NCBI, reflecting the lack of genetic information for this insect species.

### Gene Ontology (GO) Analysis

**Figure 2:**
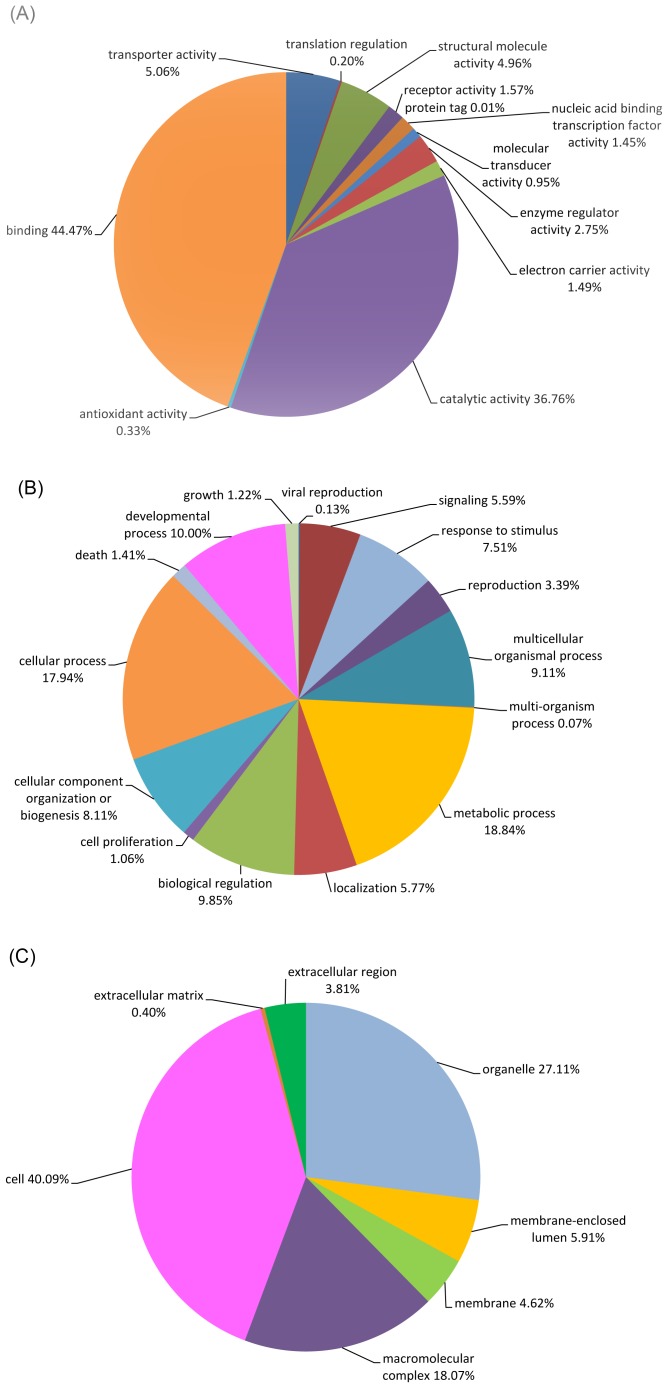
GO terms (level 2) distribution of *B.* *oleae* transcriptome. (A) molecular function, (B) biological process, (C) cellular component.

Gene Ontology (GO) terms were used for the functional categorization of the 5,426 predicted *B. oleae* proteins (38.2% of the total number of contigs). Data are shown in Table S2. In most cases more than one term was mapped to the same predicted protein. 10,098 terms for biological process categories, 3,662 for molecular function categories and 4,234 for cellular component categories were emerged. The sequences were categorized to 12 molecular function, 15 biological process and 7 cellular component categories in GO level 2 (general function categories) (Figure 2).

The majority of the molecular function GO terms were involved in binding (3,133 sequences, 44%), followed by catalytic activity (2,592 sequences, 36.76%), transporter activity (357 sequences, 5.06%), structural molecule activity (357 sequences, 4.96%), enzyme regulator activity (194 sequences, 2.75%), receptor activity (111 sequences, 1.57%), electron carrier activity (105 sequences, 1.49%), nucleic acid bindind transcription factor activity (102 sequences, 1.45%), molecular transducer activity (67 sequences, 0.95%), antioxidant activity (23 sequences, 0.33%), translation regulation (14 sequences, 0.20%) and protein tag (1 sequence, 0.01%) (Figure 2A).

Most of the biological process GO terms were involved in metabolic process (3,064 sequences, 18.84%), followed by cellular process (2,917 sequences, 17.94%), developmental process (1,626 sequences, 10.00%), biological regulation (1,601 sequences, 9.85%), multicellular process (1,482 sequences, 9.11%), cellular component organization or biogenesis (1,319 sequences, 8.11%), response to stimulus (1,221 sequences, 7.51%), localization (938 sequences, 5.77%), signaling (909 sequences, 5.59%), reproduction (551 sequences, 3.39%), death (230 sequences, 1.41%), growth (199 sequences, 1.22%), cell proliferation (172 sequences, 1.06%), viral reproduction (21 sequences, 0.13%) and multi-organism process (11 sequences, 0.07%) (Figure 2B).

Cellular component GO terms are distributed in cell (3,333 sequences, 40.09%), organelle (2,254 sequences, 27.11%), macromolecular complex (1,502 sequences, 18.07%), membrane-enclosed lumen (491 sequences, 5.91%), membrane (384 sequences, 4.62%), extracellular region (317 sequences, 3.81%) and extracellular matrix (33 sequences, 0.40%) (Figure 2C).

The GO classification results are in line with the recently sequenced transcriptomes of *B. dorsalis* [12], [13], [14], *T. vaporariorum* [15] and *Musca domestica* [16] where binding, cell and metabolic processes were the three largest groups, suggesting that 454 pyrosequencing technology provided a comprehensive representation of the *B. oleae* transcriptome. Although another Tephritid species, the Mediterranean fruit fly *Ceratitis capitata*, may also be related to *B. oleae*, a comparison with that species is not possible this time, as the only published genomic dataset for *C. capitata* contains a small number of of ESTs derived from adult head and embryos [17], which is of limited use for annotation studies, such as the one conducted here.

### Enzyme Classification and KEGG Pathway Analysis

**Figure 3:**
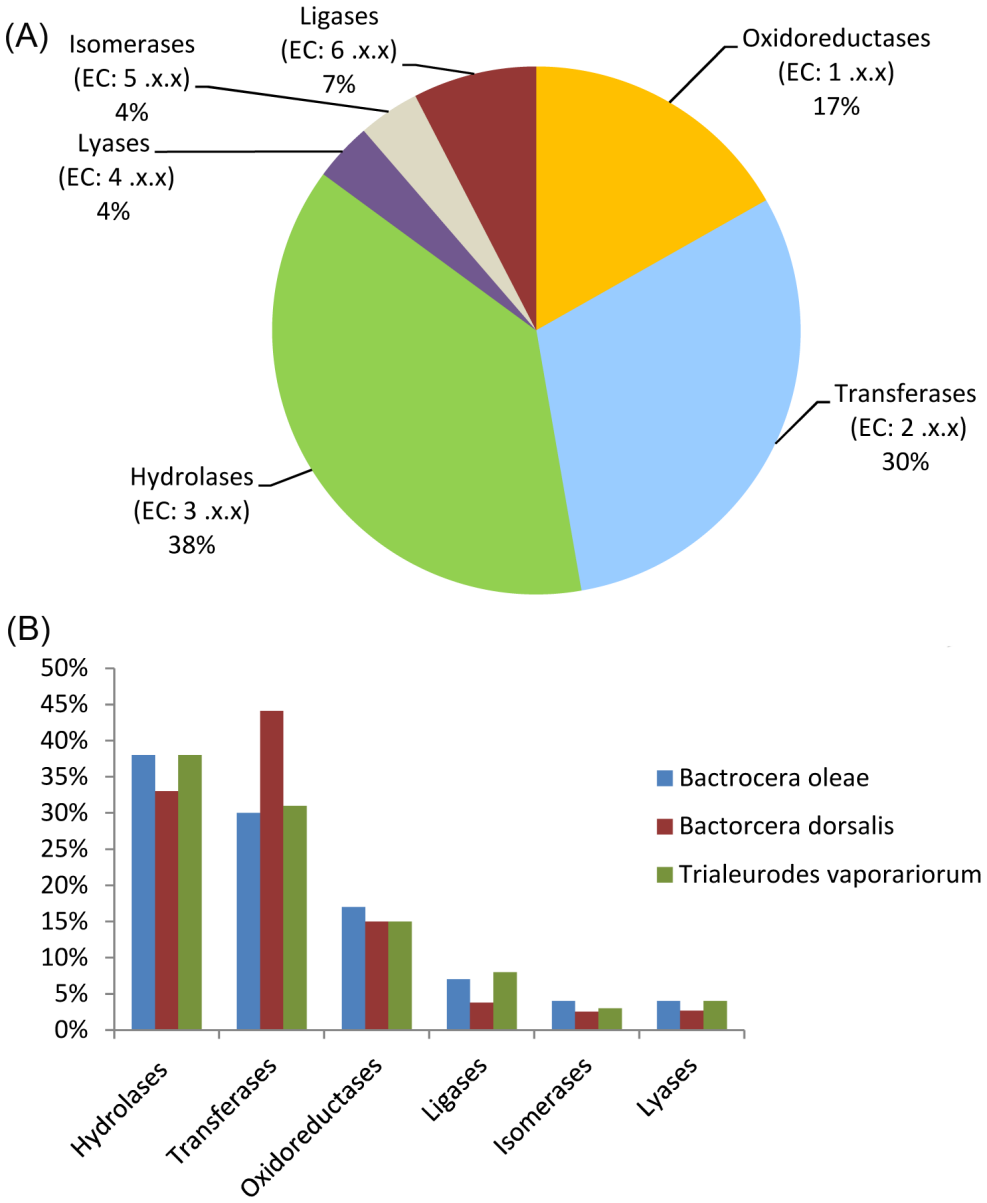
Enzyme Classification (EC) analysis of the transcriptome of ***B. oleae.*** (A) Distribution of EC number in general EC terms, (B) percentage of EC number distribution of *B. oleae* compared to that from transcriptome sequencing of *B. dorsali*s [14], *M. domestica* [16] and *T. vaporariorum* [15].

After initial bioinformatic analysis of gene functions the potential enzymes were further characterized based on the chemical reaction they catalyze. This was achieved using the predictions of Enzyme Commission (EC) numbers for each sequence. Enzyme classification revealed that hydrolases are the largest group of *B. oleae* enzymes (38%, 644 enzymes), followed by transferases (30%, 519 enzymes), oxidoreductases (17%, 286 enzymes), ligases (7%, 129 enzymes), isomerases (4%, 64 enzymes) and lyases (4%, 61 enzymes). (Figure 3A). The distribution/relative proportion of each enzyme category is similar to that determined in the white fly *T. vaporariorum* [15] and *B. dorsalis* [14] transciptomes, with the possible exception of transferases and hydrolases, which were slightly over- and under- represented respectively (Figure 3B). The 1,700 sequences having EC numbers were further characterized by Kyoto Encyclopedia of Genes and Genomes (KEGG) pathway analysis. The predicted enzymes are distributed in 122 pathways (Table S3). The best matches of KEGG mapping are presented in Table 2. Interestingly, a large number of contigs were found to be associated with drug metabolism (58 with the drug metabolism-cytochrome P450 pathway; 52 with the drug metabolism-other enzymes pathway), possibly indicating the evolution of a multi-gene system in *B. oleae*, which might have a role in xenobiotic/phytotoxin detoxification.

**Table 2:** Summary of KEGG pathway mapping of *B. oleae* contigs.

Pathway ID	Pathway	Number of contigs in Pathway
230	Purine metabolism	127
190	Oxidative phosphorylation	101
910	Nitrogen metabolism	91
240	Pyrimidine metabolism	79
480	Glutathione metabolism	61
982	Drug metabolism - cytochrome P450	58
980	Metabolism of xenobiotics by cytochrome P450	58
520	Amino sugar and nucleotide sugar metabolism	56
983	Drug metabolism - other enzymes	52
10	Glycolysis/Gluconeogenesis	47
500	Starch and sucrose metabolism	47
627	Aminobenzoate degradation	44
330	Arginine and proline metabolism	43
260	Glycine, serine and threonine metabolism	42
620	Pyruvate metabolism	40
970	Aminoacyl-tRNA biosynthesis	40
561	Glycerolipid metabolism	39
71	Fatty acid metabolism	35

### Transcripts Encoding Putative P450s

P450s are one of the largest super-families, playing a dominant role in plant-insect interactions and insecticide/xenobiotic metabolism. They are divided in 4 major clades, named CYP2, CYP3, CYP4 and mitochondrial. Eighty-eight P450s have been identified in the genome of *Drosophila melanogaster* [18], [19], while 90 P450s were found in the transcriptome of the closely related *B. dorsalis* [12], [14]. A total of 55 contigs were identified as P450 encoding genes in the *B. oleae* transcriptome (Table S4), and no allelic variants were found among P450 protein sequences encoded. Five additional P450 sequences (Table S5) were obtained by a degenerate PCR study.

The total number of P450s identified in the *B. oleae* transcriptome is similar to those found in the genomes of *D. melanogaster* and *B. dorsalis* but much lower than those of other dipteran species like e.g. *Aedes aegypti* and *Culex quinquefasciatus* having 160 and 170 P450 genes respectively [12], [14], [19], [20], [21] (Table 3). However, it is possible that additional P450 genes may await discovery in *B. oleae* genome. Notably, only one P450 sequence of *B. oleae* was available in the NCBI database prior to the present study.

**Table 3:** Comparison of P450s in different insect species.*

Cytochrome P450 clan	Bactroceraoleae	Bactroceradorsalis	Drosophilamelanogaster	Anophelesgambiae	Aedesaegypti	Apismellifera	Triboliumcastaneum	Myzus persicae	Trialeurodesvaporariorum
**CYP2**	2	6	7	10	12	8	8	3	3
**CYP3**	28	50	36	40	82	28	72	63	34
**CYP4**	17	30	32	46	57	4	45	48	13
**Mitochondrial**	13	4	11	9	9	6	9	1	7
**Total P450s**	**60**	**90**	**88**	**105**	**160**	**46**	**134**	**115**	**57**

* numbers were derived from [14], [15], [19] and this study.

**Figure 4:**
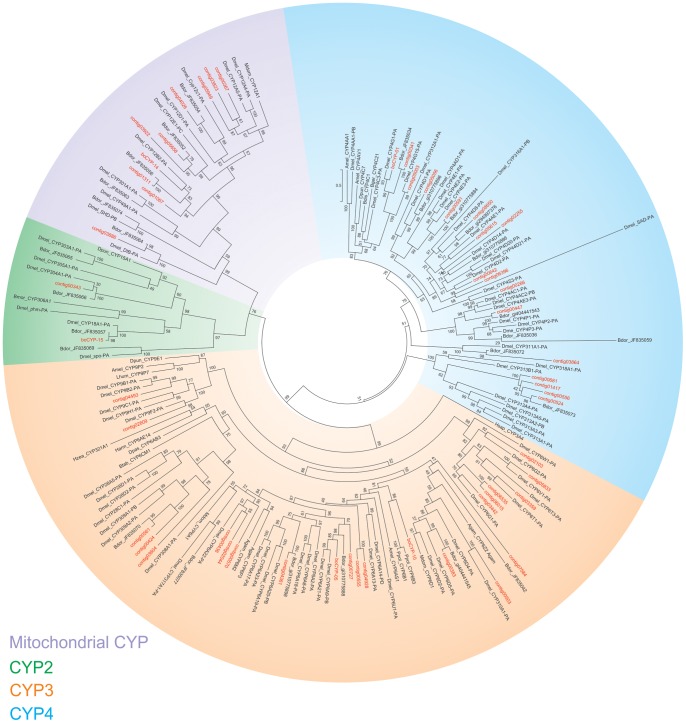
Phylogenetic analysis of ***B. oleae***putative P450s. *B. oleae* P450s clustered within the 4 major insect CYP clades. Aech: *Acromyrmex echinatior,* Agam: *Anopheles gambiae*, Amel: *Apis mellifera,* Bdor: *Bactrocera dorsalis* (“JF” sequences were obtained from Shen *et al*., 2011), Bger: *Blatella germanica,* Bmor: *Bombyx mor*i, Btab: *Bemisia tabaci*, Dmel: *Drosophila melanogaster,* Dpas: *Depressaria pastinacella*, Dpun*: Diploptera punctata*, Harm: *Helicoverpa armigera*, Hsap*: Homo sapiens*, Hzea: *Helicoverpa zea*, Mdom: *Musca domestica*, Ppol: *Papilio polyxenes*.

Based on phylogenetic analysis with other known insect P450s or the identification of closest blastp hits in the NCBI nr database in the case of misaligning protein sequences, the *B. oleae* P450s were assigned to appropriate P450 clades and families. Representatives of all 4 major insect P450 clades were found in this dataset. The majority of *B. oleae* P450s (28 out of 55) identified in this study belong to the CYP3 clade, 2 to the CYP2, 17 to the CYP4 and 13 to the mitochondrial clade (Figure 4).

Members of the CYP3 and CYP4 clades in other insect species are most commonly involved in environmental response/detoxifying functions against xeniobiotics and phytotoxins in other insects [19], [22], and the over-representation of members of those clades possibly indicates an enhanced defense mechanism present in *B. oleae* against such compounds. Orthologues of major insecticide detoxification genes in other species, such as the *Bemisia tabaci* CYP6CM1, the first P450 from an agricultural pest that was demonstrated that is capable to detoxify neonicotinoid insecticides [23], and the *D. melanogaster* CYP4G1, an insect-specific P450 oxidative decarbonylase for cuticular hydrocarbon biosynthesis [24] were among the list of genes that were identified in this study (Figure 4).

To identify P450 genes of *B. oleae* undergoing positive selection, and thus possibly playing a role switching from feeding on decaying substrates to fresh ones, a dN/dS (ω) analysis in *B. oleae*/*B. dorsalis* ortholog pairs was performed (File S1).

### Transcripts Encoding Putative GSTs

The GST super-family has also been involved in the resistance to phytotoxins and insecticides (reviewed [7]). Insect GSTs belong to seven classes, named delta (δ), epsilon (ε), omega (ω), sigma (σ), theta (θ), zeta (ζ) and microsomal GSTs. Thirty seven GSTs have been identified in *D. melanogaster* (reviewed in [25]) while 42 putative GSTs have been identified in the transcriptome of *B. dorsalis* [14]. A total of 43 contigs encoding GSTs were identified in the *B. oleae* transcriptome (Table S6). Three genes with amino-acid homology 99% at the protein level and two additional ones with identical sequences at the amino-acid level but several silent SNPs, which might represent putative allelic variants were found among these sequences: contig07254, contig08723, contig09900 are allelic variants of contig07812, contig08608 and contig09901, respectively, and contigs 09162 and 09177 are identical.

**Figure 5:**
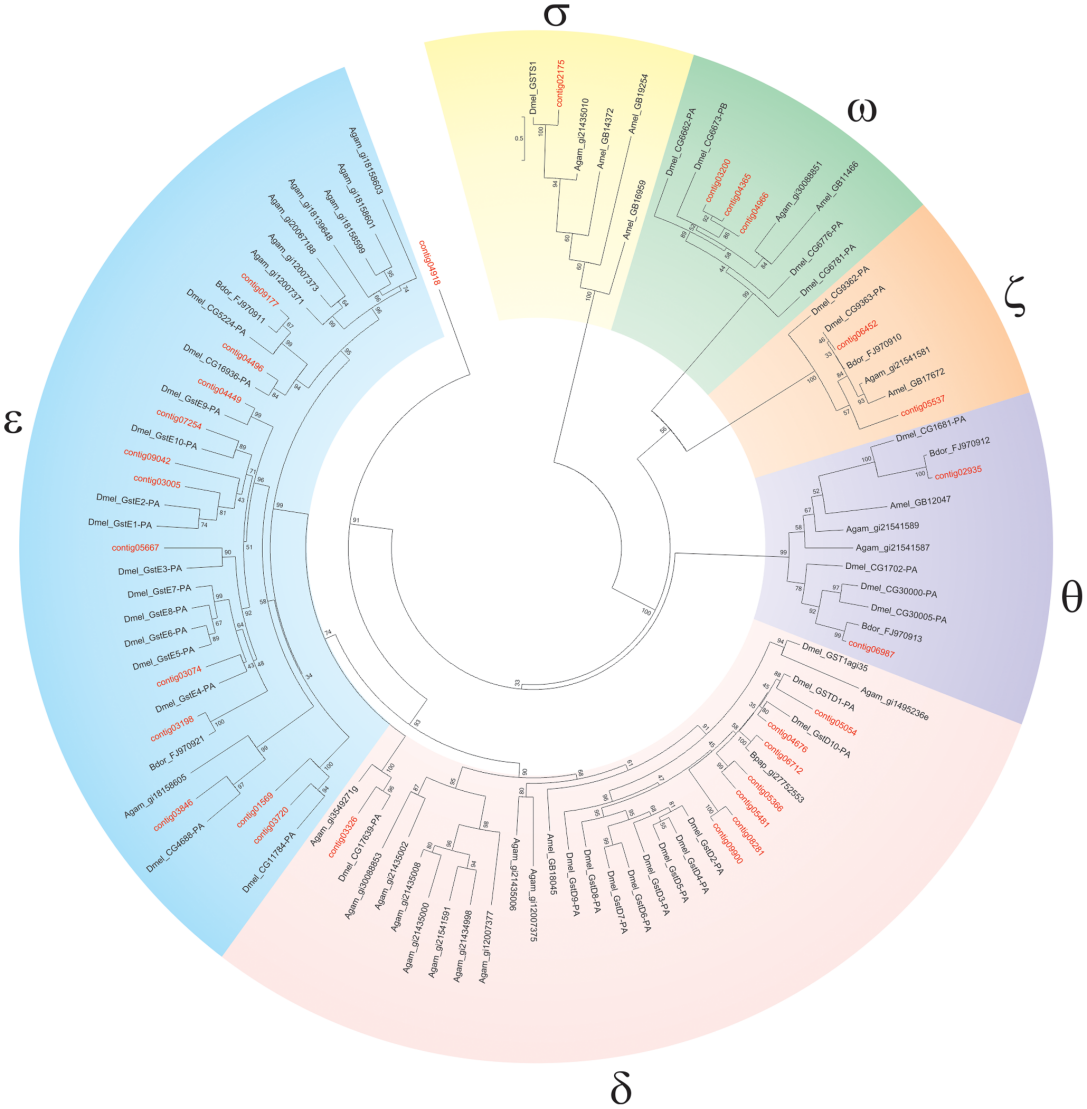
Phylogenetic analysis of *B. oleae putative GSTs*. *B. oleae* sequences, corresponding to cytosolic GSTs, clustered within classes. δ: delta class, ε: epsilon class, ζ: zeta class, θ: theta class, σ: sigma class, ω: omega class. Agam: *Anopheles gambiae*, Amel: *Apis mellifera*, Bdor: *Bactrocera dorsalis* [12], Bpap: *Bactrocera papaya*.

Based on phylogenetic analysis with other known insect GSTs or the identification of closest blastp hits in the NCBI nr protein database in the case of misaligning protein sequences, *B. oleae* GSTs were assigned to the delta, epsilon, omega, sigma, theta zeta and microsomal classes: out of 39 unique GSTs, 8 belong to the delta class, 12 to the epsilon class, 3 to omega, 1 to sigma, 4 to theta, 3 to zeta and 6 to microsomal class (Figure 5). The remaining 2 GSTs are described as delta/epsilon since they could not be assigned particularly to delta or epsilon GST class. A comparative summary of the cytosolic GSTs identified in *B. oleae* transcriptome versus those identified in other insect species is presented in Table 4. The number and distribution of cytosolic GSTs within classes in *B. oleae* is similar to that of other Diptera, such as *D. melanogaster* (reviewed in [25]) and *A. gambiae* [26], with the possible exception of epsilon GSTs which are overrepresented in *B. oleae* (Table 4). The delta and epsilon GST classes are unique in insect species and seem to be implicated in xenobiotic detoxification [20]. For example, GSTE2 of *A. gambiae* (Agam_gi12007373 in Figure 5), a glutathione transferase with DDTase activity, is responsible for conferring DDT resistance in *Anopheles gambiae* [27]. More than half (22 out of 43) of GSTs identified in the transcriptome of *B. oleae* belong to delta and epsilon classes, which might indicate an enhanced potential for xenobiotic metabolism.

**Table 4:** Comparison of cytosolic GSTs in different insect species.*

Cytosolic GST class	Bactroceraoleae	Bactrocera dorsalis	Drosophilamelanogaster	Anophelesgambiae	Aedesaegypti	Apismellifera	Triboliumcastaneum	Myzuspersicae	Trialeurodesvaporariorum
**Delta**	8	14	11	12	8	1	3	8	9
**Epsilon**	12	7	14	8	8	−	19	−	1
**Omega**	3	6	5	1	1	1	4	−	−
**Sigma**	1	1	1	1	1	4	7	8	5
**Theta**	4	3	4	2	4	1	1	2	−
**Zeta**	3	−	2	1	1	1	1	−	1
**Other**	−	1	−	3	3	−	−	−	
**Delta/Epsilon**	2	6	−	−	−	−	−	−	−
**Total cytosolic GSTs**	**33**	**39**	**37**	**28**	**27**	**8**	**35**	**18**	**16**

* numbers were derived from [14], [15], [40], [41] and this study.

To identify GST encoding genes of *B. oleae* undergoing positive selection, and thus possibly playing a role switching from feeding on decaying substrates to fresh ones, a dN/dS (ω) analysis in *B. oleae/B. dorsalis* ortholog pairs was performed (File S1).

### Transcripts Encoding Putative Carboxylesterases (CCEs)

CCEs have been shown to be involved in the detoxification of insecticides as well as the metabolism of plant derived allelochemicals (reviewed in [7]). The CCEs can be divided into 13 clades [20], [36], including acetylcholinesterases (AChE). These clades can in turn be organized into 3 classes, i.e. the dietary detoxification enzymes (clades A–C), the generally secreted enzymes (clades D–G) and the neurodevelopmental CCEs (clades I–M). Thirty-five CCEs have been identified in the genome of *D. melanogaster* (reviewed in [28]) while recently, 38 putative CCEs have been identified in the transcriptome of *B. dorsalis* [14]. A total of 15 contigs putatively encoding CCEs were identified in *B. oleae* transcriptome and no allelic variants were found among these sequences (Table S7).

**Figure 6:**
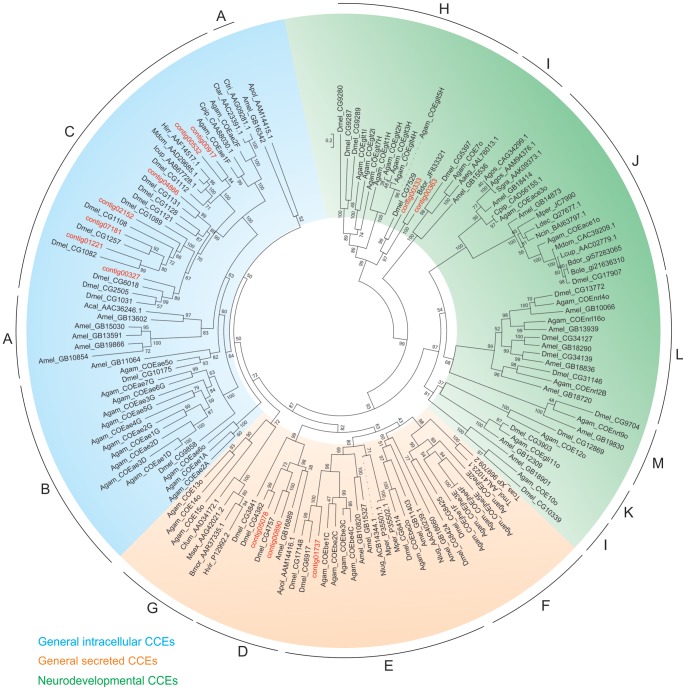
Phylogenetic analysis of *B oleae* putative CCEs. *B. oleae* CCEs clustered within clades [36]. A: hymenopteran radiation with related group containing odorant degrading esterases, B: dipteran mitochondrial, cytosolic and secreted esterases, C: dipteran microsomal, α-esterases, D: integument esterases, E: β-esterases and pheromone esterases, F: dipteran juvenile hormone esterases, G: lepidopteran juvenile hormone esterases, H: glutactin and glutactin-like enzymes, I: uncharacterized group, J: acetylcholinesterases, K: gliotactins, L: neuroligins, M: neurotactins. Aaeg: *Aedes aegypti*, Acal: *Anisopteromalus calandrae*, Agam: *Anopheles gambiae*, Agos: *Aphis gossypii*, Amel: *Apis mellifera*, Apol: *Antheraea polyphemus*, Bdor: *Bactrocera dorsalis*, Bmor: *Bombyx mori*, Bole: *Bactrocera oleae*, Cfum: *Choristoneura fumiferana*, Cpip: *Culex pipiens*, Ctar: *Culex tarsalis*, Ctri: *Culex tritaeniorhynchus*, Dmel: *Drosophila melanogaster*, Hirr: *Haematobia irritans*, Hvir: *Heliothis virescens*, Lcup: *Lucilia cuprina*, Ldec: *Leptinotarsa decemlineata*, Mdom: *Musca domestica*, Mper: *Myzus persicae,* Nlug: *Nilaparvata lugens*, Sgra: *Schizaphis graminum*, Tcas: *Tribolium castaneum,* Tmol: *Tenebrio molitor*.

Based on phylogenetic analysis with other known insect CCEs or the identification of closest blastp hits in the NCBI nr database in the case of misaligning or short CCE protein sequences, CCEs were assigned to respective clades and classes. Representatives of dipteran microsomal α-esterases (C clade), integument esterases (D clade), β-esterases and pheromone esterases (E clade) and glutactins and glutactin-like enzymes (H clade) were found in this dataset. Out of the 15 identified CCEs, 7 belong to the C clade, 2 to D clade, 1 to E clade and 2 to H clade (Figure 6). The remaining 3 CCEs could not be assigned to any particular CCE clade (Table S7). Comparative analysis (Table 5) with CCEs from other known insect species shows that the number of identified CCEs is considerably less than those from other insects. However, the majority of them (7 out of 15) are assigned to the dietary class, which might indicate a possible association of this gene super-family with the ability of olive fly to cope with substances present in the olive flesh.

**Table 5:** Comparison of CCEs in different insect species.*

Carboxyl/cholinesterases**	Bactroceraoleae	Bactroceradorsalis	Drosophilamelanogaster	Anophelesgambiae	Aedesaegypti	Apismellifera	Triboliumcastaneum	Myzuspersicae	Trialeurodesvaporariorum
**Dietary class**		19							
**A clade**	−		−	−	−	5	−	5	11
**B clade**	−		13	16	22	3	14	−	−
**C clade**	7		−	−	−	−	12	−	1
**Hormone/semiochemical processing**		1							
**D clade**	2		3	−	−	1	2	−	−
**E clade**	1		2	4	2	2	7	12	6
**F clade**	−		3	6	6	2	2	−	−
**G clade**	−		0	4	6	−	−	−	−
**Neurodevelopmental**		4							
**H clade**	2		5	10	7	1	1	−	1
**I clade**	−		1	1	1	1	1	1	1
**J clade**	−		1	2	2	2	2	3	2
**K clade**	−		1	1	1	1	1	1	1
**L clade**	−		4	5	5	5	5	−	3
**M clade**	−		2	2	2	1	2	−	1
**Unclassified**	3	14	−	−	−	−	−	−	−
**Total CCEs**	**15**	**38**	**35**	**51**	**54**	**24**	**49**	**21**	**27**

* numbers were derived from [14], [15], [40], [41] and this study.

** for full CCE clade names, see legend of Figure 6.

To identify CCE enconding genes of *B. oleae* undergoing positive selection, and thus possibly playing a role switching from feeding on decaying substrates to fresh ones, a dN/dS (ω) analysis in *B. oleae*/*B. dorsalis* ortholog pairs was performed (File S1).

### Transcripts Encoding Putative ABC Transporters

The ATP-binding cassete (ABC) transporter superfamily is considered to play a major role in the ability of insects to cope with xenobiotics [29]. Fifty six ABC transporters have been identified in *D. melanogaster* (reviewed in [30]). A total of 18 contigs encoding ABCs were identified in the *B. oleae* transcriptome (Table S8). No allelic variants were found among those sequences. Based on phylogenetic analysis or, in the case where the nucleotide binding domain (NBD) was missing, based on the closest blastp hits in the NCBI nr database, ABC transporters were assigned to different subfamilies. The number of identified ABC transporters is less than those from other insects [29]. Out of 18 *B. oleae* ABCs, four belong to the B subfamily of which only two are full transporters (those clustering with hsABCB1) (Figure S2), two to the C subfamily and four to the G subfamily. Interestingly, these families are believed to be the most relevant to xenobiotic detoxification [29] and are about half of the ABC transporters identified in this study. Furthermore, three ABC transporters belong to the A-family, one to the D subfamily, one to the E subfamily and three to the F subfamily (Figure S3 and S4). A comparative summary of the ABCs identified in *B. oleae* transcriptome versus those identified in other insect species is presented in Table 6.

**Table 6:** Comparison of ABC genes in different insect species.*

ABC subfamily	Bactroceraoleae	Drosophilamelanogaster	Anophelesgambiae	Apismellifera	Triboliumcastaneum
**A**	3	10	9	3	9
**B**	4	8	5	7	6
**C**	2	14	13	9	31
**D**	1	2	2	2	2
**E**	1	1	1	1	1
**F**	3	3	3	3	3
**G**	4	15	16	15	13
**H**	−	3	3	3	3
**Total ABC transporters**	**18**	**56**	**52**	**43**	**68**

* numbers were derived from [42], [43] and this study.

### Conclusions

We have generated, using 454 pyrosequencing technology, a *B. oleae* transcriptome dataset containing 14,204 contigs. This dataset represents a very significant expansion of the number of cDNA sequences currently available for *B. oleae*. Although the function of the majority of the assembled sequences is unknown, it is likely that new ongoing projects will facilitate their future annotation and our efforts to understand their role in the physiology and fundamental biology of the olive fruit fly.

We have identified and phylogenetically classified at least 132 putative major detoxification genes (60 P450s; 39 GSTs; 15 CCEs; 18 ABC transporters) involved in the metabolism of xenobiotics, such as plant phytotoxins and insecticides.

These new data and genomic resources developed in this study for *B. oleae* will be useful to the community studying this significant crop pest and will substantially facilitate molecular studies of underlying mechanisms involved in insecticide resistance and adaptation of *B. oleae* larvae in olive fruits as well as other important aspects of olive fruit fly biology. As our understanding of the regulation of detoxification mechanisms increase, new strategies should be devised for the development of more efficient, eco-friendly and species-specific strategies for pest control.

## Materials and Methods

### Insect Sample and mRNA Isolation

In order to obtain a large and broad transcriptome data set, RNA was extracted from a pool of different life stages of *B. oleae*, including mixed lab strains (Vontas et al 2002) and field caught insects (collected from Herakleion, Crete in 2011–2012) fed on artificial diet and olives (equal representation in each stage), with a proportion: 10 eggs; 4 instar larva, 3 pupae; 4 adults (two males and two females, 4 and 20 day old). This pooled sample was snap frozen in liquid nitrogen and used for mRNA isolation, using an mRNA-Only Eukaryotic mRNA isolation kit (Epicente, USA).

The study was carried out on private land of University of Crete and the owner of the land gave permission to conduct the study on this site and collect insects from olive trees present in this land. No specific permissions were required for these locations/activities, since the study only involved collection of a few insects for sequencing analysis, from abandoned olive fruit fly populations widespread in Crete. The field studies did not involve endangered or protected species.

### cDNA Library Preparation, Sequencing and Assembly

cDNA synthesis and amplification was performed using Mint-Universal cDNA Synthesis kit (Evrogen, Russia) and 1 μg of *B. oleae* mRNA. About 800 ng of amplified cDNA were used as starting material in the normalization reaction using the Trimmer kit (Evrogen, Russia). Normalized material was re-amplified for 18 cycles and subsequently digested with 10 Units SfiI for 2 hours at 48°C. Fragments larger than 800 bp were isolated from a Low Melting Point agarose gel and purified using the MinElute Gel Extraction kit (Qiagen, Germany). Purified cDNA fragments (200 ng) were ligated into 100 ng of a dephoshorylated pDNR-lib Vector, pre-digested with SfiI (Clontech, USA) using the Fast Ligation kit (New England Biolabs, USA). Ligations were desalted by ethanol precipitation and re-dissolved in 10 μl water. Three-fold desalted ligation was used to transform NEB10b competent cells (New England Biolabs, USA).

Roughly a million clones were plated on LB-Cm plates, scrapped off the plates and stored as glycerol stocks at −70°C. One half of the cells was used to inoculate a 300 ml Terrific Broth/Cm culture, which was grown for 5 hours at 30°C. Plasmid DNA was prepared using standard methods (Qiagen, Germany). Purified plasmid DNA (200 μg) was digested with 100 Units SfiI for 2 hours at 48°C. cDNA inserts were gel-purified (LMP- Agarose/MinElute Extraction kit) and ligated to high-molecular-weight DNA using a proprietary SfiI-linker.

Library generation for the 454 FLX sequencing was carried out according to the manufacturer’s standard protocols (Roche/454 life sciences, USA). In short, the concatenated inserts were sheared randomly by nebulization to fragments ranging in size from 400 to 900 bp. These fragments were end polished and the 454 A and B adaptors that are required for the emulsion PCR and sequencing were added to the ends of the fragments by ligation. The resulting fragment library was sequenced on a picotiterplate (PTP) on the GS FLX using the Roche/454 Titanium chemistry (LCG Genomics, Berlin, Germany).

Prior to assembly, the sequence reads were screened for the SfiI-linker that was used for the concatenation, the linker sequences were clipped out of the reads and the clipped reads assembled to individual transcripts using the Roche/454 Newbler software at default settings (454 Life Sciences Corporation, Software Release: 2.6). Raw sequence data and the transcriptome assembly were submitted under the BioProject accession number PRJNA195424 in the NCBI-database.

### Degenerate PCR

A degenerate PCR strategy for insect P450s [31] had been initiated prior to the transcriptomic study, to amplify orthologous regions from *B. oleae*. cDNA synthesis was carried out using the SuperScript III protocol and the PCR products were isolated, sub-cloned into pGEMT-easy vector (Promega) and sequenced in both directions. Obtained *B. oleae* P450 partial sequences were deposited in the NCBI-database (GenBank accession numbers: KC917331, KC917335, KC917340, KC917344, KC917345).

### Blast Homology Searches and Sequence Annotation

For blast homology searches and sequence annotation Blast2GO software v.2.6.0 [32] was used, as previously described for the analysis of *Manduca sexta* and *Trialeurodes vaporariorum* transcriptomes [15], [33]. For homology searches, all *B. oleae* contigs larger than 200 bp were searched using blastx against the NCBI non-redundant (nr) protein database, using an e-value cut-off of 1E^−3^. The sequences that did not give any blastx hit were subsequently searched using blastn against the NCBI nr nucleotide database using an e-value cut-off of 1E^−10^.

For gene ontology (GO) mapping, Blast2GO recovers all the GO terms associated to the hits obtained by the blast search. After the mapping step, results were subjected to GO annotation, a process of selecting GO terms from the GO pool and assigning them to the query sequences. The sequences were further annotated using InterPro. The functionality of InterPro annotation in Blast2GO allows retrieving domain/motif information in a sequence-wide manner. GO terms corresponding to these Interpro-domains, were then transferred to the sequences and merged with already existent GO terms. GO terms were modulated using the annotation augmentation tool ANNEX [34], followed by GOSlim. As described in the Blast2GO tutorial ”the ANNEX approach uses uni-vocal relationships between GO terms from different GO categories to add implicit annotation and GOSlim is a reduced version of the GO that contains a selected number of relevant nodes”. In this study, the generic GOSlim mapping term was used.

Finally, Enzyme classification (EC) codes and the Kyoto Encyclopedia of Genes and Genomes (KEGG) metabolic pathway annotations were obtained through the direct mapping of GO terms to the corresponding enzyme codes.

### Analysis of Genes Related to Xenobiotic Detoxification

*B. oleae* contigs (>200 bp) encoding P450s, GSTs, CCEs and ABC transporters were identified using tblastn (E-value cutoff <1E^−3^) and *Drosophila melanogaster* and *Bactrocera dorsalis* protein sequences of P450S, GSTs, CCEs and ABCs as query (*D. melanogaster* sequences were downloaded from GenBank and *B. dorsalis* sequences were taken from [12]). Each contig encoding a detoxification gene was manually curated for frame shifts and sequencing errors. Contigs were translated and trimmed using BioEdit version 7.1.9 and searched by blastx (E-value <1E^−3^) against all the assembled contigs. Results with more than 99% similarity were considered as allelic variants.

Muscle 3.8.31 [35] was used to perform multiple sequence alignment of *B. oleae* P450, GST, CCE or ABC protein sequences with a representative dataset of their counterparts in other species (File S2). Unfortunately, not many detoxification genes of the closely related *B. dorsalis* could be included in our alignment as at the time of writing mainly raw sequence data (Sequence Read Archives) of this species was deposited in the NCBI database [12], [13], [14].

For each detoxification family, only protein sequences showing no misalignment were used in the final alignment for phylogenetic analysis. Since N- and C-termini of CCEs are variable, all CCE protein sequences were trimmed as previously described in [36]. Only the nucleotide binding domains (NBDs) of the ABC transporters were used for phylogenetic analysis. NBDs were extracted using the ScanProsite facility (http://expasy.org/tools/scanprosite) in combination with the PROSITE profile PS5089. When an ABC protein sequence contained 2 NBDs, the N-terminus NBD was selected for further analysis.

Model selection was performed with ProtTest 2.4 [37] and the optimum model for phylogenetic analysis was selected according to Akaike information criterion (P450s: LG+G+F, GSTs: LG+I+G, CCEs: LG+I+G, ABC B: LG+I+G, ABC D: LG+G, ABC EF: LG+I+G). A maximum likelihood analysis was performed using Treefinder (version of March 2011, [38]) and a bootstrap analysis with 1000 pseudoreplicates (LR-ELW) was performed to evaluate the branch strength of each tree. The resulting tree was midpoint rooted and edited with MEGA 5.0 software [39].

## Supporting Information

Figure S1 **Blast statistics.** (A) E-value and (B) percentage similarity.
(TIF)

Figure S2 **Phylogenetic analysis of** ***B*** **.** ***oleae*** **putative ABC transporters (subfamily B**). Four *B. oleae* ABC sequences are clustered in B subfamily. Agam: *Anopheles gambiae*, Amel: *Apis mellifera,* Bmor: *Bombyx mori,* [42] Dpul*: Daphnia pulex* [43], Dmel: *Drosophila melanogaster*, Hsap: *Homo sapiens*, Tcas: *Tribolium castaneum*.
(TIF)

Figure S3 **Phylogenetic analysis of** ***B. oleae*** **putative ABC transporters (subfamily D).** One *B. oleae* ABC sequence is clustered in D subfamily**.** Agam: *Anopheles gambiae*, Amel: *Apis mellifera*, Dmel: *Drosophila melanogaster*, Dpul: *Daphnia pulex*, Hsap: *Homo sapiens*.
(TIF)

Figure S4 **Phylogenetic analysis of** ***B. oleae*** **putative ABC transporters (subfamilies E and F**). One *B. oleae* ABC sequence is clustered in E and one in F subfamily. Agam: *Anopheles gambiae*, Amel: *Apis mellifera,* Bmor: *Bombyx mori,* Dmel: *Drosophila melanogaster*, Dpul*: Daphnia pulex*, Hsap: *Homo sapiens*, Tcas: *Tribolium castaneum.*
(TIF)

Table S1 **Top BLAST hits in the NCBI nr database.** Only contigs with a blast hit are presented.
(XLSX)

Table S2 **Gene ontology categorization results.** Only contigs which returned one or more GO terms are presented.
(XLSX)

Table S3 **Distribution of putative enzymes in KEGG pathways.**
(XLSX)

Table S4 **Sequence name, nucleotide and amino acid length and sequence, clade and clade identification method of contigs encoding for putative** ***B. oleae*** **P450s.**
(XLSX)

Table S5 **Sequence name, accession number, nucleotide and amino acid length and sequence, clade and clade identification method of** ***B. oleae*** **P450s obtained by degenerate PCR.**
(XLSX)

Table S6 **Sequence name, nucleotide and amino acid length and sequence, class and class identification method of contigs encoding for putative** ***B. oleae*** **GSTs.**
(XLSX)

Table S7 **Sequence name, nucleotide and amino acid length and sequence, clade and clade identification method of contigs encoding for putative** ***B. oleae*** **CCEs.**
(XLSX)

Table S8 **Sequence name, nucleotide and amino acid length and sequence, subfamily and subfamily identification method of contigs encoding for putative** ***B. oleae*** **ABC transporters.**
(XLSX)

File S1 **dN/dS (ω) analysis in** ***B. oleae*** **/** ***B. dorsalis*** **ortholog pairs (P450s, GSTs, CCEs).**
(DOCX)

File S2 **Multiple alignment of** ***B. oleae*** **P450, GST, CCE and ABC transporters (B, D, E and F subfamilies) protein sequences with a representative dataset of their counterparts in other species.** Aaeg: *Aedes aegypti*, Acal: *Anisopteromalus calandrae*, Aech: *Acromyrmex echinatior*, Agam: *Anopheles gambiae*, Agos: *Aphis gossypii*, Amel: *Apis mellifera,* Apol: *Antheraea polyphemus,* Bdor: *Bactrocera dorsalis* (“JF” sequences obtained from [12]), Bger: *Blatella germanica,* Bmor: *Bombyx mori*, Bole: *Bactrocera oleae,* Bpap: *Bactrocera papaya* Btab: *Bemisia tabaci*, Cfum: *Choristoneura fumiferana*, Cpip: *Culex pipiens*, Ctar: *Culex tarsalis*, Ctri: *Culex tritaeniorhynchus*, Dmel: *Drosophila melanogaster,* Dpas:*Depressaria pastinacella*, Dpul: *Daphnia pulex*, Dpun*: Diploptera punctata*, Harm: *Helicoverpa armigera*, Hirr: *Haematobia irritans*, Hsap*: Homo sapiens*, Hvir: *Heliothis virescens*, Hzea: *Helicoverpa zea*, Lcup: *Lucilia cuprina*, Ldec: *Leptinotarsa decemlineata*, Mdom: *Musca domestica*, Mper: *Myzus persicae,* Ncin: *Nephotettix cincticeps*, Nlug: *Nilaparvata lugens*, Ppol: *Papilio polyxenes*, Sgra: *Schizaphis graminum*, Tcas: *Tribolium castaneum,* Tmol: *Tenebrio molitor*.
(ZIP)

## References

[1] Daane KM, Johnson MW (2010) Olive fruit fly: Managing an ancient pest in modern times. Annu Rev Entomol 5: 155–169. 10.1146/annurev.ento.54.110807.090553 19961328

[2] Ant T, Koukidou M, Rempoulakis P, Gong H-F, Economopoulos A, et al (2012) Control of the olive fruit fly using genetics-enhanced sterile insect technique. BMC Biol 10: 51. 22713628 10.1186/1741-7007-10-51 PMC3398856

[3] Vontas J, Hernández-Crespo P, Margaritopoulos JT, Ortego F, Feng H-T, et al (2011) Insecticide resistance in Tephritid flies. Pest Biochem Physiol 100: 199–205.

[4] Vontas JG, Hejazi MJ, Hawkes NJ, Cosmidis N, Loukas M, et al (2002) Resistance-associated point mutations of organophosphate insensitive acetylcholinesterase, in the olive fruit fly *Bactrocera oleae* . Insect Mol Biol 11: 329–336. 12144698 10.1046/j.1365-2583.2002.00343.x

[5] Kakani EG, Ioannides IM, Margaritopoulos JT, Seraphides NA, Skouras PJ, et al (2008) A small deletion in the olive fly acetylcholinesterase gene associated with high levels of organophosphate resistance. Insect Biochem Mol Biol 38: 781–787. 18625401 10.1016/j.ibmb.2008.05.004

[6] Hsu J-C, Feng H-T, Wu W-J, Geib SM, Maoa C-H, et al (2012a) Truncated transcripts of nicotinic acetylcholine subunit gene Bda6 are associated with spinosad resistance in *Bactrocera dorsalis* . Insect Biochem Mol Biol 42: 806–815. 22898623 10.1016/j.ibmb.2012.07.010

[7] Li X, Schuler MA, Berenbaum MR (2007) Molecular mechanisms of metabolic resistance to synthetic and natural xenobiotics. Annu Rev Entomol 52: 231–253. 16925478 10.1146/annurev.ento.51.110104.151104

[8] Dermauw W, Wybouw N, Rombauts S, Menten B, Vontas J, et al (2012) A link between host plant adaptation and pesticide resistance in the polyphagous spider mite *Tetranychus urticae* . Proc Natl Acad Sci U S A 110: 393–394. 10.1073/pnas.1213214110 PMC3545796 23248300

[9] Corrado G, Alagna F, Rocco M, Renzone G, Varricchio P, et al (2012) Molecular interactions between the olive and the fruit fly *Bactrocera oleae.* . BMC Plant Biol 12: 86. 22694925 10.1186/1471-2229-12-86 PMC3733423

[10] Hagen KS (1966) Dependence of the olive fruit fly *Dacus oleae* larvae on symbiosis with *Pseudomonas savastanoi* for the utilization of olive. Nature 209: 423–424.

[11] Tsoumani KT, Augustinos AA, Kakani EG, Drosopoulou E, Mavragani-Tsipidou P, et al (2011) Isolation, annotation and applications of expressed sequence tags from the olive fly, *Bactrocera oleae* . Mol Genet Genomics 285: 33–45. 20978910 10.1007/s00438-010-0583-y

[12] Shen G-M, Dou W, Niu J-Z, Jiang H-B, Yang W-J, et al (2011) Transcriptome analysis of the oriental fruit fly (*Bactrocera dorsalis*). PLoS One 6: e29127. 22195006 10.1371/journal.pone.0029127 PMC3240649

[13] Zheng W, Peng T, He W, Zhang H (2012) High-throughput sequencing to reveal genes involved in reproduction and development in *Bactrocera dorsalis* (Diptera: Tephritidae). PLoS One 7: e36463. 22570719 10.1371/journal.pone.0036463 PMC3343016

[14] Hsu J-C, Chien T-Y, Hu C-C, May Chen M-J, Wu W-J, et al (2012b) Discovery of genes related to insecticide resistance in *Bactrocera dorsalis* by functional genomic analysis of a *de novo* assembled transcriptome. PLoS One 7: e40950. 22879883 10.1371/journal.pone.0040950 PMC3413685

[15] Karatolos N, Pauchet Y, Wilkinson P, Chauhan R, Denholm I, et al (2011) Pyrosequencing the transcriptome of the greenhouse whitefly, *Trialeurodes vaporariorum* reveals multiple transcripts encoding insecticide targets and detoxifying enzymes. BMC Genomics 12: 56. 21261962 10.1186/1471-2164-12-56 PMC3036619

[16] Liu F, Tang T, Sun L, Jose Priya TA (2012) Transcriptomic analysis of the housefly (*Musca domestica*) larva using parallel pyrosequencing. Mol Biol Rep 39: 1927–1934. 21643958 10.1007/s11033-011-0939-3

[17] Gomulski LM, Dimopoulos G, Xi Z, Soares MB, Bonaldo MF, et al (2008) Gene discovery in an invasive tephritid model pest species, the Mediterranean fruit fly, Ceratitis capitata. BMC genomics 9: 243. 18500975 10.1186/1471-2164-9-243 PMC2427042

[18] Tijet N, Helvig C, Feyereisen R (2001) The cytochrome P450 gene superfamily in *Drosophila melanogaster*: Annotation, intron–exon organization and phylogeny. Gene 262: 189–198. 11179683 10.1016/s0378-1119(00)00533-3

[19] Feyereisen R (2011) Insect CYP Genes and P450 Enzymes. Insect Mol Biol Biochem (ed L.I. Gilbert) Ch. 8: 236–316 (Elsevier 2011)..

[20] Ranson H, Claudianos C, Ortelli F, Abgrall C, Hemingway J, et al (2002) Evolution of supergene families associated with insecticide resistance. Science 298: 179–181. 12364796 10.1126/science.1076781

[21] Strode C, Wondji CS, David JP, Hawkes NJ, Lumjuan N, et al (2008) Genomic analysis of detoxification genes in the mosquito *Aedes aegypti* . Insect Biochem Mol Biol 38: 113–123. 18070670 10.1016/j.ibmb.2007.09.007

[22] Berenbaum MR (2002) Postgenomic chemical ecology: From genetic code to ecological interactions. J Chem Ecol 28: 873–896. 12049229 10.1023/a:1015260931034

[23] Karunker I, Bentinq J, Lueke B, Ponge T, Nauen R, et al (2008) Over-expression of cytochrome P450 CYP6CM1 is associated with high resistance to imidacloprid in the B and Q biotypes of *Bemisia tabaci* (Hemiptera: Aleyrodidae). Insect Biochem Mol Biol 38: 634–644. 18510975 10.1016/j.ibmb.2008.03.008

[24] Qiu Y, Tittiger C, Wicker-Thomas C, Le Goff G, Young S, et al (2012) An insect-specific P450 oxidative decarbonylase for cuticular hydrocarbon biosynthesis Proc Natl Acad Sci USA. 11 109: 14858–63. 10.1073/pnas.1208650109 PMC3443174 22927409

[25] Tu CP, Akgü B (2005) *Drosophila* glutathione S-transferases. Methods Enzymol 401: 204. 16399388 10.1016/S0076-6879(05)01013-X

[26] Ding Y, Ortelli F, Rossiter LC, Hemingway J, Ranson H (2003) The *Anopheles gambiae* glutathione transferase supergene family: annotation, phylogeny and expression profiles. BMC Genomics 4: 35. 12914673 10.1186/1471-2164-4-35 PMC194574

[27] Ranson H, Rossiter L, Ortelli F, Jensen B, Wang X, et al (2001) Identification of novel class of insect glutathione S-transferases involved in resistance to DDT in the malaria vector *Anopheles gambiae* . Biochem J 359: 295–304. 11583575 10.1042/0264-6021:3590295 PMC1222147

[28] Oakeshott JG, Claudianos C, Campbell PM, Newcomb RD, Russel RJ (2005) Biochemical genetics and genomics of insect esterases. Compreh Mol Insect Science-Pharmacology Vol.5 (editors Gilbert LI, Iatrou K and Gill SS) 309–381 Elsevier Oxford.

[29] Labbe R, Caveney S, Donly C (2011) Genetic analysis of the xenobiotic resistance associated ABC gene subfamilies of the Lepidoptera. Insect Mol Biol 20: 243–256. 21199020 10.1111/j.1365-2583.2010.01064.x

[30] Dassa E, Bouige P (2001) The ABC of ABCs: a phylogenetic and functional classification of ABC systems in living organisms. Res Microbiol 152: 211–229. 11421270 10.1016/s0923-2508(01)01194-9

[31] Snyder MJ, Scott JA, Andersen JF, Feyereisen R (1996) Sampling P450 diversity by cloning polymerase chain reaction products obtained with degenerate primers. Methods Enzymol 272: 304–12. 8791790 10.1016/s0076-6879(96)72036-0

[32] Conesa A, Götz S, García-Gómez JM, Terol J, Talón M, et al (2005) Blast2GO: a universal tool for annotation, visualization and analysis in functional genomics research. Bioinformatics 21: 3674–3676. 16081474 10.1093/bioinformatics/bti610

[33] Pauchet Y, Wilkinson P, Vogel H, Nelson DR, Reynolds SE, et al (2010) Pyrosequencing the *Manduca sexta* larval midgut transcriptome: messages for digestion, detoxification and defence. Insect Mol Biol 19: 61–75. 19909380 10.1111/j.1365-2583.2009.00936.x

[34] Myhre S, Tveit H, Mollestad T, Laegreid A (2006) Additional gene ontology structure for improved biological reasoning. Bioinformatics 22: 2020–2027. 16787968 10.1093/bioinformatics/btl334

[35] Edgar RC (2004) MUSCLE: multiple sequence alignment with high accuracy and high throughput. Nucleic Acids Res 32: 1792–1797. 15034147 10.1093/nar/gkh340 PMC390337

[36] Claudianos C, Ranson H, Johnson RM, Biswas S, Schuler MA, et al (2006) † A deficit of detoxification enzymes: pesticide sensitivity and environmental response in the honeybee. Insect Mol Biol 15: 615–636. 17069637 10.1111/j.1365-2583.2006.00672.x PMC1761136

[37] Abascal F, Zardoya R, Posada D (2005) ProtTest: selection of best-fit models of protein evolution. Bioinformatics 21: 2104–2105. 15647292 10.1093/bioinformatics/bti263

[38] Jobb G, von Haeseler A, Strimmer K (2004) TREEFINDER: a powerful graphical analysis environment for molecular phylogenetics. BMC Evol Biol 4: 18. 15222900 10.1186/1471-2148-4-18 PMC459214

[39] Tamura K, Peterson D, Peterson N, Stecher G, Nei M, et al (2011) MEGA5: Molecular Evolutionary Genetics Analysis Using Maximum Likelihood, Evolutionary Distance, and Maximum Parsimony Methods. Mol Biol Evol 28: 2731–2739. 21546353 10.1093/molbev/msr121 PMC3203626

[40] Hayes JD, Flanagan JU, Jowsey IR (2005) Glutathione transferases. Annu Rev Pharmacol Toxicol 45: 51–88. 15822171 10.1146/annurev.pharmtox.45.120403.095857

[41] Oakeshott JG, Johnson RM, Berenbaum MR, Rnason H, Cristino AS, et al (2010) Metabolic enzymes associated with xenobiotic and chemosensory responses in *Nasonia vitripennis* . Insect Mol Biol 19 (Suppl. 1)147–163. 20167025 10.1111/j.1365-2583.2009.00961.x

[42] Sturm A, Cunningham P, Dean M (2009) The ABC transporter gene family of *Daphnia pulex* . BMC Genomics 10: 170. 19383151 10.1186/1471-2164-10-170 PMC2680897

[43] Liu S, Ling Tian Z, Guo E, Luan Y, Zhang J, et al (2011) Genome-wide identification and characterization of ATP-binding cassette transporters in the silkworm, *Bombyx mori* . BMC Genomics 12: 491. 21981826 10.1186/1471-2164-12-491 PMC3224256

