# Correction: Analysis of the Olive Fruit Fly *Bactrocera oleae* Transcriptome and Phylogenetic Classification of the Major Detoxification Gene Families

**DOI:** 10.1371/annotation/ab935a02-e8a2-4d7e-85cb-f8c48d6d9ba2

**Published:** 2013-09-20

**Authors:** Nena Pavlidi, Wannes Dermauw, Stephane Rombauts, Antonis Chrisargiris, Thomas Van Leeuwen, John Vontas

There were multiple errors in the Author Contributions statement. The Author Contributions statement should read: "Conceived and designed the experiments: NP JV. Performed the experiments: NP WD SR AC. Analyzed the data: NP WD SR TVL. Contributed reagents/materials/analysis tools: AC SR. Wrote the paper: NP WD JV."

In addition, a funding organization was incorrectly omitted from the Funding Statement. The following sentence should be included in the Funding Statement: "T.V.L. is a post-doctoral fellow of the Fund for Scientific Research Flanders (FWO)." 

