# Correction: Analysis of the Olive Fruit Fly *Bactrocera oleae* Transcriptome and Phylogenetic Classification of the Major Detoxification Gene Families

**DOI:** 10.1371/journal.pone.0128056

**Published:** 2015-05-08

**Authors:** The PLOS ONE Staff

The fourth author’s name is spelled incorrectly. The correct name is: Antonios Chrysargyris. The correct citation is: Pavlidi N, Dermauw W, Rombauts S, Chrysargyris A, Van Leeuwen T, Vontas J, et al. (2013) Analysis of the Olive Fruit Fly Bactrocera oleae Transcriptome and Phylogenetic Classification of the Major Detoxification Gene Families. PLoS ONE 8(6): e66533. 10.1371/journal.pone.0066533
