# Supplementary figures and images for: Analysis of the Olive Fruit Fly Bactrocera oleae Transcriptome and Phylogenetic Classification of the Major Detoxification Gene Families
Source: PLoS One. 2013 Jun 18;8(6):e66533. doi: 10.1371/journal.pone.0066533 (PMC3688913; doi:10.1371/journal.pone.0066533)

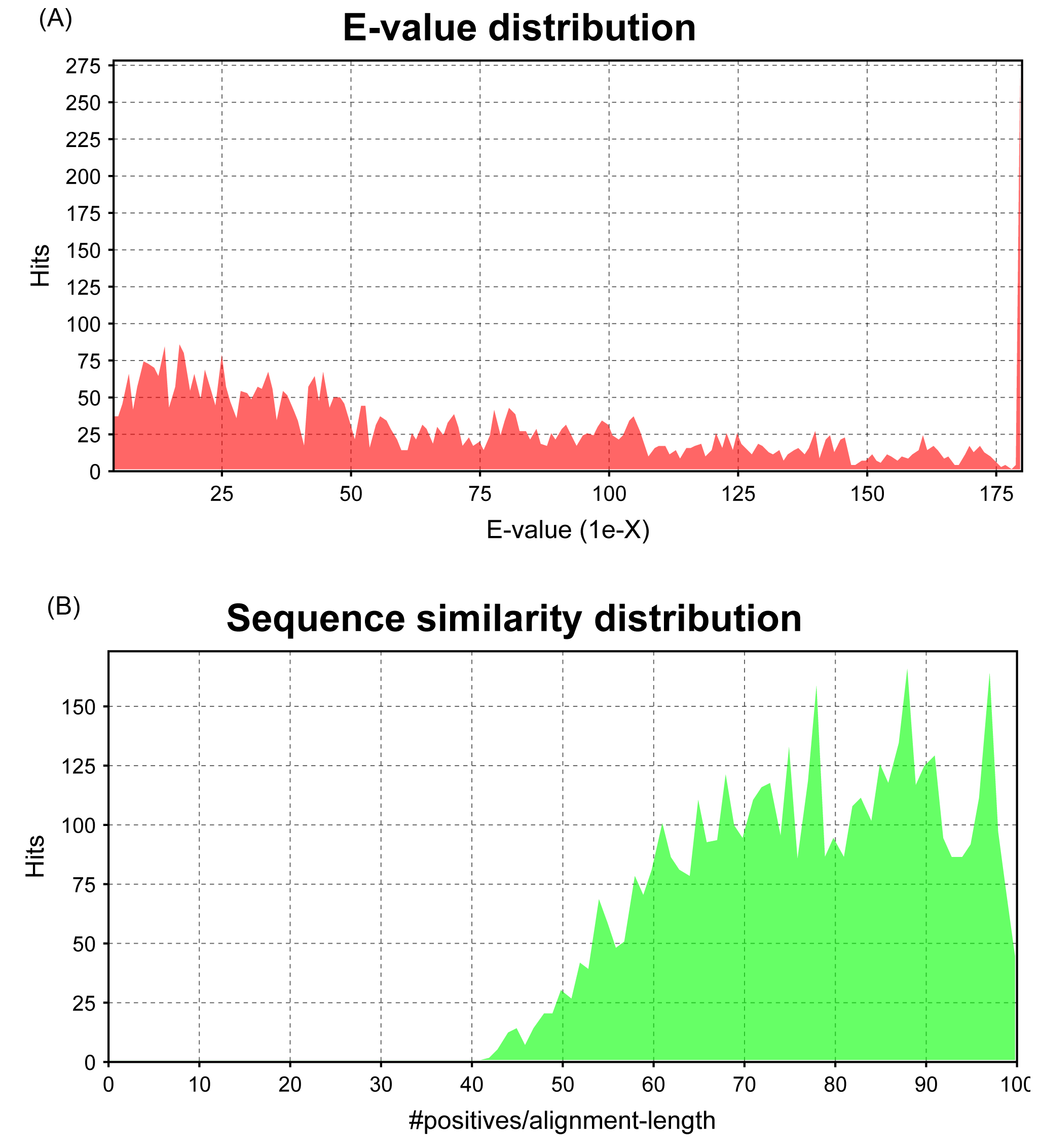

Supplement: Figure S1 — Blast statistics. (A) E-value and (B) percentage similarity. (TIF) [file pone.0066533.s001.tif]

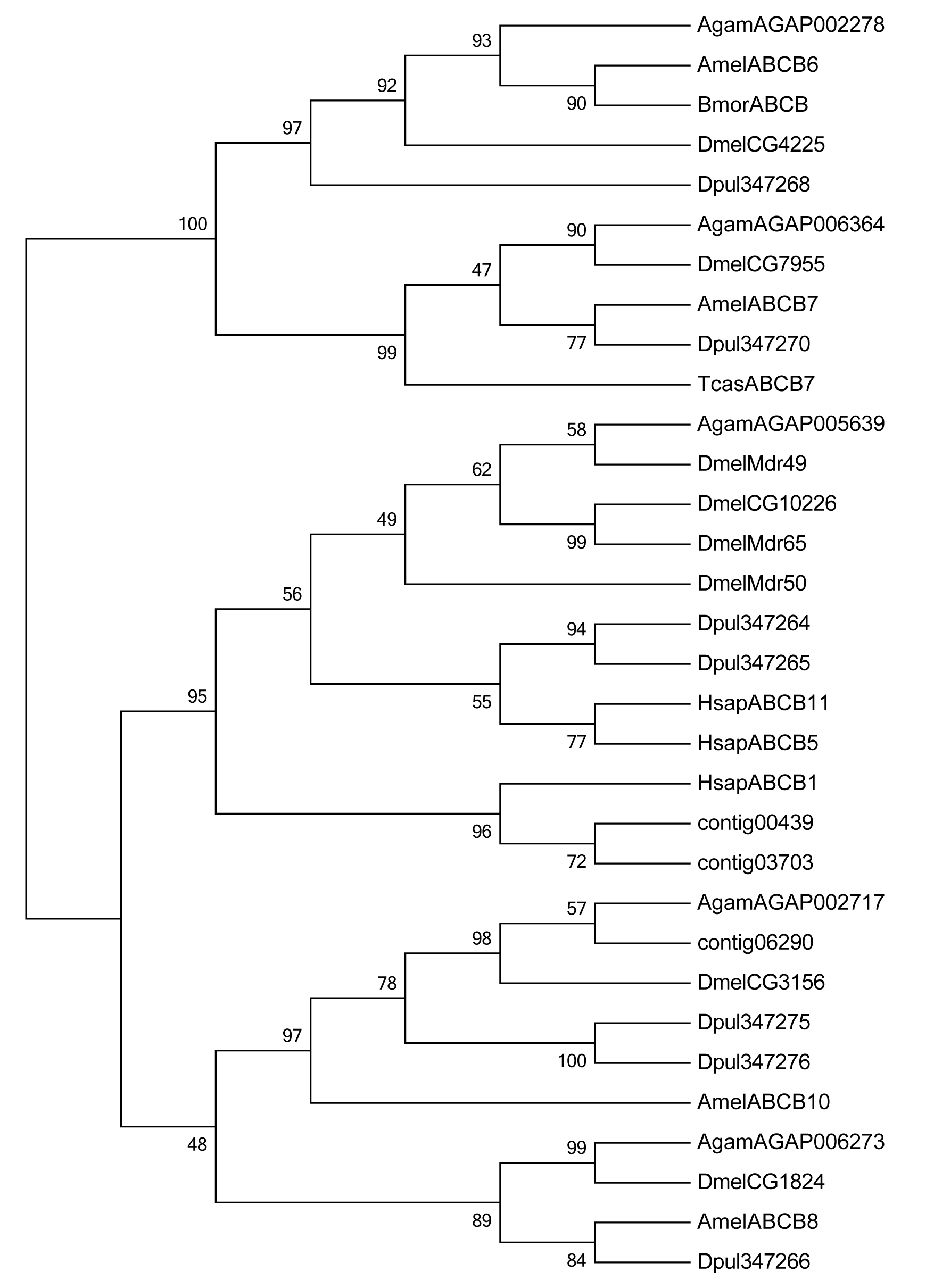

Supplement: Figure S2 — Phylogenetic analysis of B . oleae putative ABC transporters (subfamily B). Four B. oleae ABC sequences are clustered in B subfamily. Agam: Anopheles gambiae, Amel: Apis mellifera, Bmor: Bombyx mori, [42] Dpul: Daphnia pulex [43], Dmel: Drosophila melanogaster, Hsap: Homo sapiens, Tcas: Tribolium castaneum. (TIF) [file pone.0066533.s002.tif]
