# Supplementary material for: Analysis of the Olive Fruit Fly Bactrocera oleae Transcriptome and Phylogenetic Classification of the Major Detoxification Gene Families
Source: PLoS One. 2013 Jun 18;8(6):e66533. doi: 10.1371/journal.pone.0066533 (PMC3688913; doi:10.1371/journal.pone.0066533)
Supplement: File S1 — dN/dS (ω) analysis in B. oleae / B. dorsalis ortholog pairs (P450s, GSTs, CCEs). (DOCX) [file pone.0066533.s013.docx]

**Materials and methods**

*Bactrocera dorsalis* orthologs of detoxification genes (P450s, GSTs, CCEs) of *Bactrocera oleae* were identified based on our phylogenetic analyses (Fig. 4, Fig.5 and Fig. 6). Pairs having a bootstrap value greater than 70 % were considered as orthologs and were selected for further analysis. Pair-wise amino acid alignments of the region in common between two orthologs were conducted using Muscle 3.8.31 [35]. The amino acid sequences were back-translated to nucleotide sequences and used for the estimation of the pairwise non-synonymous (dN) and synonymous (dS) substitution rates using MEGA 5.05 [39]. The Jukes-Cantor distance model with the modified Nei-Gojobori method was used. The pair-wise ratios of dN/dS (ω) were calculated and used to investigate if *B. oleae* sequences were evolved under positive (ω > 1) or negative, purifying (ω < 1) selection or neutrally (ω = 1) compared to the corresponding sequences of *B. dorsalis.*

**Results and Discussion**

To identify detoxification genes of *B. oleae* undergoing positive selection and thus possibly playing a role switching from feeding on decaying substrates to fresh ones, a dN/dS (ω) analysis was performed (Table S1.1). Among the 22 pairs of *B. oleae/B. dorsalis* orthologs dN/dS values ranged from 0.672 to 1.727, with an average of 0.967. Eight pairs had a dN/dS value greater than 1. Among the sequence with a dN/dS value greater than one, two *B. oleae* contigs (contig05658 and contig0426), coding for P450 genes ranked the highest (with a dN/dS ratio of 1.727 of 1.276, respectively). This might suggest that these two P450s are under positive selection and contribute to the ability of *B. oleae* to feed on fresh olives. However, as only fragments of *Bactrocera* CYP sequences were compared and dN/dS ratio vary greatly depending on gene region (conserved versus non conserved domains) this hypothesis should be taken with care.

**Table S1.1 Summary of dN/dS analysis**

| ***B. oleae* contig** | ***B. dorsalis* ortholog** | **dN/dS** | **Length of analyzed sequence (nt)** |
| --- | --- | --- | --- |
| **P450s** |  |  |  |
| Contig05003 | Bdor_JF835034 | 0.973 | 639 |
| Contig05056 | Bdor_gi310775896 | 0.819 | 729 |
| Contig00591 | Bdor_gi310775884 | 1.044 | 792 |
| Contig08650 | Bdor_gi294997376 | 0.696 | 498 |
| Contig00447 | Bdor_gi404441543 | 0.987 | 1,557 |
| Contig03864 | Bdor_JF835072 | 0.932 | 579 |
| Contig00524 | Bdor_JF835073 | 0.744 | 513 |
| Contig00383 | Bdor_gi404441545 | 0.909 | 1,551 |
| Contig00727 | Bdor_gi310775888 | 1.135 | 1,251 |
| BoCYP-05 | Bdor_gi310775888 | 1.222 | 450 |
| Contig04361 | Bdor_gi310775898 | 0.882 | 822 |
| Contig00561 | Bdor_JF835070 | 1 | 477 |
| BoCYP-15 | Bdor_JF835057 | 1.222 | 435 |
| Contig00343 | Bdor_JF835066 | 0.840 | 459 |
| Contig03886 | Bdor_JF835064 | 0.714 | 966 |
| Contig01311 | Bdor_JF835056 | 0.672 | 720 |
| Contig05658 | Bdor_JF835052 | 1.727 | 444 |
| Contig04026 | Bdor_JF835054 | 1.276 | 765 |
| **CCEs** |  |  |  |
| Contig02935 | Bdor_JF970912 | 0.724 | 495 |
| Contig06987 | Bdor_JF970913 | 0.693 | 453 |
| Contig03198 | Bdor_JF970921 | 1.162 | 471 |
| **GSTs** |  |  |  |
| Contig00363 | Bdor_JF833321 | 0.921 | 1,086 |
